# Syncope vs. Seizure: Ictal Bradycardia and Ictal Asystole

**DOI:** 10.1155/2024/1299282

**Published:** 2024-05-06

**Authors:** Sumika Ouchida, Kaitlyn Parratt, Armin Nikpour, Greg Fairbrother

**Affiliations:** ^1^Neurology Department, Royal Prince Alfred Hospital, Sydney, NSW, Australia; ^2^The University of Sydney School of Medicine, Sydney, NSW, Australia; ^3^School of Health & Human Sciences, Southern Cross University, Sydney, NSW, Australia; ^4^Patient and Family-Centred Care Research, Sydney Local Health District, Sydney, NSW, Australia; ^5^School of Nursing and Midwifery, The University of Sydney, Sydney, NSW, Australia

## Abstract

**Background:**

Ictal arrhythmia is a rare condition that causes arrhythmic manifestations induced by epileptic seizures, including asystole or bradycardia. Ictal asystole (IA) is a very rare condition found in patients undergoing video-encephalography (EEG) monitoring. It is often related to temporal lobe epilepsy and can cause syncope, which can lead to injury or even death. *Case Presentation*. Two patients with epilepsy showed symptoms of syncope. Both patients underwent 4-day ambulatory EEG tests and were diagnosed with IA. Following the tests, the patients were implanted with a permanent pacemaker, and one of them underwent a temporal lobectomy. As a result of these procedures, the patients experienced a reduction in episodes of symptomatic syncope.

**Conclusion:**

Patients with ictal asystole and symptomatic ictal bradycardia are at increased risk of falls due to seizures. Although there are no specific guidelines for managing this condition, antiseizure medications, epilepsy surgery, and cardiac pacemaker implantation have been effective treatments.

## 1. Introduction

Epileptic seizures are caused by excessive synchronised neural activity and can result in sensory, motor, and behavioural manifestations with or without loss of consciousness. In most patients, seizures cause temporary changes in cardiorespiratory function, resulting in tachycardia, tachypnea, apnea, and bradycardia, which may lead to secondary syncope [1]. Cardiac arrhythmias have been frequently observed in association with epileptic seizures. Ictal asystole (IA) is a rare condition found in 0.27–0.4% of patients undergoing video electroencephalography (EEG) monitoring [2, 3]. IA is defined as the absence of ventricular complexes for >4 seconds accompanied by electrographic seizure onset [4], and ictal bradycardia (IB) is defined as an R–R interval greater than 2 seconds [5]. Usually, IA and IB are related to temporal lobe epilepsy and can result in syncope, accompanied by injury or death [1]. Cardiac events have been suggested as possible reasons for sudden unexplained death in epilepsy (SUDEP). SUDEP describes the sudden and unexpected death of a person with epilepsy, where no apparent cause can be identified during postmortem examination, such as trauma or status epilepticus [6].

There is limited information about IA, which is when the heart stops during a seizure. To date, no in-depth analysis has been published on the clinical features of this condition in comparison to seizures without asystole [1]. Descriptions of observed episodes of IA are rare. The most common cardiac changes seen during seizures are ictal tachycardia (IT) and ictal bradycardia. Changes in blood pressure often occur. The typical course of an IA event is an initial sinus tachycardia followed by a progressive decrease in heart rate, which may or may not be present at the beginning of the seizure. This decrease can lead to asystole of variable duration [7]. The cause of ictal-induced bradyarrhythmia is not well understood. Cardiovascular autonomic control arises from specific areas of the brain, such as the insular cortex, amygdala, cingulate gyrus, hypothalamus, brainstem, and prefrontal cortex, which regulate changes in heart rate and blood pressure. Asystole may be caused by transmitting ictal activity from the temporal lobe to the adjacent insula. This could cause a slowing of the heart rate, and its connections to subcortical areas may contribute to ictal changes in heart rate [8, 9]. This is known as the “lock-step phenomenon,” which refers to the synchronisation between the central autonomic centres and the postganglionic autonomic activity. The phenomenon happens when there is a time-locked relationship between postganglionic cardiac sympathetic discharge and cortical epileptiform activity. It has been suggested that ictal activity may lead to changes in heart rate by increasing vagal tone, as changes in heart rate often follow epileptic activity [8].

Clinical signs and symptoms: During a seizure, the heart rate usually increases at the start— sinus tachycardia. Subsequently, the heart rate gradually decreases, sometimes leading to a complete absence of heart activity— asystole. The decrease in heart rate may or may not be present from the beginning of the seizure. During asystole, the seizure signs may persist, or the seizures may be interrupted by one or more symptoms associated with syncope, such as complete loss of consciousness, atonia, fall, and myoclonic jerks. It may be hard to differentiate the loss of consciousness from signs of a focal seizure with loss of awareness. In focal seizures with loss of awareness, the altered consciousness takes the form of decreased awareness and motor arrest, during which the patient is motionless and unresponsive. The patient may or may not exhibit other symptoms or signs of a focal seizure before experiencing a loss of consciousness. However, the distinction between loss of consciousness and awareness can be subjective [10]. Following the events, the patient often experiences prolonged confusion and exhaustion. It is important to note that IA can occur in the seizure onset from temporal, frontal, and insular cortical areas, which can cause diagnostic confusion and may contribute to SUDEP [11].

It has been observed that the risk of new-onset IA may increase in females with pre-existing heart conditions, especially if focal epileptic activity is also present, even in cases where the epilepsy is otherwise benign [12]. Late-onset IA typically results from neuronal network changes in therapy-resistant epilepsy. This condition seems more prevalent in males, with accompanying autonomic dysregulation as a contributing factor [12]. The following case studies provide clinical insight into IA/IB. The patients have given written consent for sharing and publication of their information.

## 2. Case Report

### 2.1. Case 1

The patient was a 75-year-old right-handed male with a history of drug-resistant focal epilepsy who presented for evaluation of events of syncope for several months. He was diagnosed with focal epilepsy at the age of 40 when he had a focal to bilateral tonic-clonic seizure. According to his partner, he began making a growling noise and frothing at the mouth. He then became stiff and collapsed to the floor, convulsing and biting his tongue. The episode lasted one minute, followed by several minutes of confusion and drowsiness. He experienced focal seizures, which began with a tingling sensation in his head, followed by speech arrest, impaired awareness, and aimless wandering with oral and manual automatisms. The seizure episode lasted for a minute and was followed by confusion and agitation. He had experienced frequent seizures for many years and then developed new episodes of collapse accompanied by loss of consciousness. His partner reported that he would become pale while walking and then suddenly fall, losing consciousness for a brief period and then become groggy and unresponsive. He was confused and agitated for about 10–30 minutes until he could sleep. The duration of these events ranged from 30 seconds to a minute.

**Figure 1:**
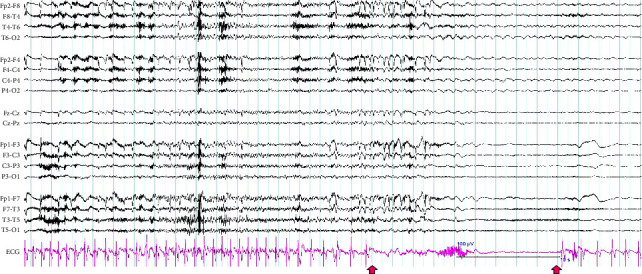
During the synchronous recording of electroencephalography (EEG) and echocardiogram (ECG) using bipolar montages (double banana), the patient experienced a left temporal seizure and asystole. The ECG lead showed a complete AV block emerging in the pink lead, and the arrow indicated an 18-second asystole on the ECG lead at the end of the seizure. The ECG gradually returned to normal sinus rhythm afterwards.

He had a history of hypercholesterolemia, asthma, and focal epilepsy. He regularly took levetiracetam 1000 mg twice daily, lamotrigine 200 mg twice daily, rosuvastatin 20 mg daily, and ventolin inhaler as needed. He had no family history of epilepsy or seizures. He was a retired nonsmoker who drank socially and lived with his partner. The patient had several medical tests, including a 12-lead ECG, a 24-hour Holter monitor, and a stress test. All the results from these tests were normal. A magnetic resonance imaging (MRI) of his brain did not show any abnormalities, but a Positron emission tomography (PET) scan revealed hypometabolism in the left anterior temporal region. The patient underwent prolonged ambulatory EEG (AEEG) tests for four days to gather more information. During the test, he reported three focal seizures during day time. The test captured a total of five seizures, including three that were reported and two subclinical seizures during his sleep. All the seizures originated from the left temporal lobe region and were accompanied by a prolonged sinus arrest that lasted for 18 seconds (as shown in Figure 1).

A repeat Holter monitor test was performed immediately after the ambulatory EEG test, confirmed peri ictal sinus arrest lasting 10–18 seconds. He was diagnosed with IA. Due to high-frequency seizures and injuries resulting from syncope due to asystole, he had a permanent pacemaker (PPM) implanted. He continued to have focal seizures monthly but no further syncope. His repeated AEEG tests in 2019 and 2023 showed no interictal epileptiform activity.

### 2.2. Case 2

The patient was a 66-year-old, right-handed female who had a history of drug-resistant focal epilepsy. She presented with increased frequency of two different types of events. According to her partner, the first type of event involved sudden onset fidgeting or rubbing with her fingers and lips, followed by smacking and unresponsiveness that lasted 2-3 minutes. After the event, confusion persisted for 5–10 minutes. The second type of event involved a sudden onset drop attack, where she fell backward and lost consciousness, remaining unresponsive for 3–8 minutes. Afterwards, confusion persisted for up to 20 minutes. She was incontinent of urine. The patient had no recollection of either event. These events happened monthly.

**Figure 2:**
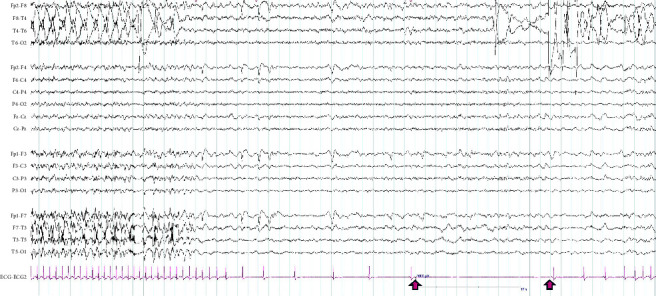
During the synchronous recording of electroencephalography (EEG) and echocardiogram (ECG) using bipolar montages (double banana), the patient experienced a left temporal seizure and asystole. The ECG lead showed a complete AV block emerging in the pink lead, and the arrow indicated a 12-second asystole on the ECG lead at the end of the seizure. The ECG gradually returned to normal sinus rhythm afterwards.

The patient had a medical history of hypertension, myocardial infarction with stents, cone biopsy due to carpel tunnel, a cyst in the left hippocampus, and multiple head strikes associated with drop attacks. She did not have a family history of epilepsy or seizure disorder and did not experience childhood febrile convulsions. She was prescribed lamotrigine 100 mg twice daily, carbamazepine 400 mg twice daily, levetiracetam 1500 mg twice daily, metoprolol 50 mg daily, lisinopril 10 mg daily, rosuvastatin 20 mg daily, and aspirin 100 mg daily. She lived in a mobile home with her partner and was retired. Clinical examination was normal. The 12-lead ECG and the routine EEG test were both unremarkable. The PET scan of the brain revealed hypometabolism in the left temporal region, while an MRI revealed the presence of a cyst in the left hippocampus. To further investigate, the patient underwent a prolonged AEEG test for four days, which captured one seizure during day time originating from the left temporal region. The seizure was accompanied by a prolonged sinus arrest that lasted for 13 seconds (as shown in Figure 2).

**Figure 3:**
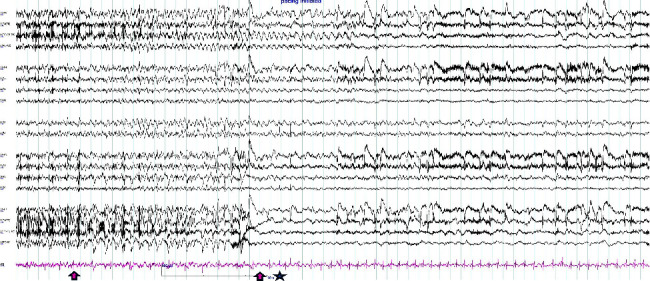
While performing synchronous recording of electroencephalography (EEG) and echocardiogram (ECG) with bipolar montages (double banana), the patient had a left temporal seizure and developed a bradycardia rhythm. The ECG lead displayed a bradycardia rhythm in the pink lead, and an arrow indicated a bradycardia rhythm on the ECG lead at the end of the seizure. The pacemaker kicked in, as denoted by the star mark. The ECG gradually returned to normal sinus rhythm afterwards.

She was diagnosed with IA. A permanent pacemaker was inserted to prevent further symptomatic events related to prolonged sinus arrest associated with her focal seizures. Episodes of collapse resolved following PPM insertion, while typical focal seizures persisted. The patient underwent inpatient video EEG monitoring for 5 days for presurgical evaluation. She had seven daytime stereotypical seziures during her monitoring, and there was a seizure with bradycardia rhythm on the ECG tracing before the pacing maker initiation (as shown in Figure 3). After the surgical evaluation, she underwent a left temporal lobectomy with prolonged seizure freedom.

## 3. Discussion

The diagnosis of IA among epilepsy patients can be challenging, and there is still controversy surrounding the optimal management strategies. This is due to the overlap in symptoms that can resemble both cardiology and neurology issues. In addition, syncope in a patient with epilepsy can be challenging to diagnose and treat. Frequent episodes of losing consciousness can be common. Making an accurate diagnosis mainly depends on the descriptions provided by the patient and witnesses of the events. Though additional tests may often be required, they are likely to yield normal results unless an episode is captured during video EEG monitoring. Relying solely on the EEG or ECG recording may lead to incorrect conclusions and inappropriate therapy being started [8]. Most patients are not suspected of having an IA or IB condition until they experience a clinical event captured during prolonged video EEG/ECG monitoring. Distinguishing between cerebrogenic and cardiogenic causes of cardiac arrhythmias can be challenging. However, simultaneous EEG/ECG recording can help differentiate a primary cardiac arrhythmia from a secondary central arrhythmia. Lim et al. have emphasised that the simultaneous recording of EEG and ECG is necessary to distinguish between causes. In both cases, the primary cerebrogenic nature of bradyarrhythmia was convincingly demonstrated, with EEG seizure activity occurring before the onset of bradyarrhythmia. When evaluating patients with epilepsy or syncope, it is essential to rule out any underlying heart conditions [13]. A cardiac investigation, including 24 hour Holter ECG monitoring, should be performed. However, performing prolonged video EEG/ECG monitoring on all patients with epilepsy or syncope may be impractical. Ambulatory EEG is a cost-effective alternative for inpatient video-EEG monitoring. It provides continuous and simultaneous EEG/ECG monitoring in real-life settings, which may be more convenient and acceptable to the patient. Compared to inpatient monitoring, ambulatory EEG is more affordable and flexible [14]. The recent commercial availability of ambulatory video EEG (aVEEG) monitoring provides valuable information. Prolonged ambulatory EEG, with or without video testing, is a practical and effective method for capturing IA/IB during seizures.

Implantable loop recorders (ILRs) are devices used to investigate cardiac arrhythmia and unexplained syncope. They are invasive and costly but have shown effective in diagnosing syncope in some one-eighth of patients previously diagnosed with epilepsy [15]. ILRs have also been shown to confirm epilepsy diagnosis in a small case series [15, 16]. ILRs have also been shown to be useful for patients with infrequent seizures [15]. Wearable external loop recorders (ELRs) such as “HeartBug” are also useful in investigating episodes of unexplained syncope. They continuously monitor cardiac rhythm and save events triggered by the patient or in response to auto triggers such as brady or tachyarrhythmias [16]. If AEEG tests are unavailable in medical institutions, ILR and ELR are alternatives for detecting cardiac arrhythmia episodes. However, IB or IA cannot be diagnosed without an EEG.

IB and IA predominantly occur during focal seizures with loss of awareness. It has been observed that there is a high incidence of comorbidity between epilepsy and obstructive sleep apnea (OSA) [17]. The exact underlying pathophysiology of this co-occurrence remains poorly understood. People with OSA are at risk of developing cardiac arrhythmia, including bradyarrhythmia. The exact mechanism of OSA-induced bradyarrhythmia is not fully understood. Lack of oxygen significantly contributes to the development of bradycardia and asystole. It has been hypothesised that an increase in parasympathetic tone may be the underlying cause of asystole during OSA episodes [17]. The use of continuous positive airway pressure (CPAP) can significantly reduce bradyarrhythmia events [18]. Therefore, clinicians should complete their examination with aVEEG recording to avoid misinterpreting epileptic seizures as apneas.

There are currently no established guidelines for managing ictal arrhythmias. Treatment is not recommended for symptomatic ictal bradyarrhythmia. A recent algorithm developed by Sowden et al. may assist in diagnosing and managing patients suspected of IA [16]. While antiseizure medications and surgical interventions have been shown to reduce ictal arrhythmias effectively, the use of cardiac pacemakers is still debated. It is recommended to use pacemakers more selectively, especially when ASMs are ineffective, patients are ineligible for epilepsy surgery or have had unsuccessful surgery [3, 10], [19]. Conclusive evidence of linkage between SUDEP and IA/IB is often difficult to obtain [20]. Drug-resistant epilepsy is a risk factor for both IA and SUDEP, but there is no evidence to support IA as a direct cause of SUDEP [3]. Most SUDEP cases are linked to generalise seizures, which typically do not cause slow heart rhythms. Although seizures can decrease heart rate, it is generally not severe, and SUDEP appears to be triggered by postictal cardiorespiratory failure [21].

Although the exact pathophysiological mechanism is not clearly defined, some studies suggest that most cases of SUDEP result from central respiratory dysfunction after a seizure, ultimately leading to cardiac arrest [22]. SUDEP is a major cause of premature mortality in epilepsy patients; therefore, it is crucial to understand how seizures impact cardiovascular function.

## 4. Conclusion

IB and IA are rare conditions. Treatments such as ASMs, epilepsy surgery, and cardiac pacemakers can effectively manage their symptoms. Cardiac pacemakers can significantly reduce the risk of seizure-related falls and loss of consciousness in some patients. Cardiac pacing has been proven to be a safe treatment option for patients who experience persistent seizures. This treatment does not worsen seizure frequency or duration or lead to generalised tonic-clonic seizures. If a patient is suspected of having ictal asystole, it is recommended to perform concurrent EEG-ECG monitoring such as ambulatory EEG testing, optimise ASMs, and consider epilepsy surgery, if feasible. Using ILRs/ELRs may aid in diagnosing this condition. If a patient experiences frequent fainting episodes accompanied by ictal asystole lasting longer than 6 seconds, even with medical treatment, it is advisable to consider a permanent pacemaker to reduce the risk of complications. Updating current guidelines to incorporate the latest research findings on this condition is essential.

## Data Availability

All data generated or analysed during this study are included in this article and are available from the corresponding author upon reasonable request.
